# Medium- to long-term health condition of patients post-COVID-19, exercise intolerance and potential mechanisms: A narrative review and perspective

**DOI:** 10.1177/20503121241296701

**Published:** 2024-11-08

**Authors:** Fabian Schwendinger, Denis Infanger, Debbie J Maurer, Thomas Radtke, Justin Carrard, Julia M Kröpfl, Aglaia Emmenegger, Henner Hanssen, Christoph Hauser, Udo Schwehr, Hans H Hirsch, Julijana Ivanisevic, Karoline Leuzinger, Aurélien E Martinez, Marc Maurer, Thomas Sigrist, Lukas Streese, Roland von Känel, Timo Hinrichs, Arno Schmidt-Trucksäss

**Affiliations:** 1 Division of Sports and Exercise Medicine, Department of Sport, Exercise and Health, University of Basel, Basel, Switzerland; 2 Faculty of Medicine, University of Basel, Basel, Switzerland; 3 Epidemiology, Biostatistics and Prevention Institute, University of Zurich, Zürich, Switzerland; 4 Clinical Virology, University Hospital Basel, Basel, Switzerland; 5 Transplantation and Clinical Virology, Department Biomedicine, University of Basel, Basel, Switzerland; 6 Metabolomics Platform, Faculty of Biology and Medicine, University of Lausanne, Quartier UNIL-CHUV, Lausanne, Switzerland; 7 Division of Infectious Diseases and Hospital Epidemiology, University Hospital Basel, Basel, Switzerland; 8 Department of Pneumology, Cantonal Hospital Olten, Olten, Switzerland; 9 Department of Pulmonology, Clinic Barmelweid, Barmelweid, Switzerland; 10 Department of Consultation-Liaison Psychiatry and Psychosomatic Medicine, University Hospital Zurich, University of Zurich, Zurich, Switzerland; 11 Department of Clinical Research, University Hospital Basel, University of Basel, Basel, Switzerland

**Keywords:** Maximum oxygen uptake, endothelial function, post-COVID-19 syndrome, long-COVID

## Abstract

**Background:**

Patients recovering from COVID-19 often present with impaired health and persisting symptoms such as exercise intolerance ⩾3 months post-infection. Uncertainty remains about long-term recovery. We aimed to review studies examining cardiac function, macro- or microvascular function, blood biomarkers and physical activity in adult patients post-COVID-19 and highlight current knowledge gaps.

**Results:**

Using echocardiography, persistent cardiac involvement of the left ventricle was observed in a fraction of patients both hospitalized and non-hospitalized. Ventricular dysfunction was often subclinical but may partly contribute to exercise intolerance post-COVID-19. Endothelial dysfunction was seen on micro- and macrovascular levels using retinal vessel imaging methods and brachial artery flow-mediated dilation, respectively. Studies reporting blood biomarkers of disease-specific impairment and endothelial dysfunction yielded upregulated inflammation, hypercoagulability, organ and endothelial damage up to several months after infection. Omics’ scale lipid profiling studies provide preliminary evidence of alterations in several lipid subspecies, mostly during acute COVID-19, which might contribute to subsequent endothelial and cardiometabolic dysfunction. Yet, more robust evidence is warranted. Physical activity may be reduced up to 6 months post-COVID-19. However, studies measuring physical activity more precisely using accelerometry are sparse. Overall, there is growing evidence for long-term multiple organ dysfunction.

**Conclusion:**

Research combining all the above methods in the search for underlying mechanisms of post-COVID-19 symptoms is mostly missing. Moreover, studies with longer follow-ups (i.e. ⩾18 months) and well-matched control groups are lacking. The findings may aid the development of rehabilitation regimes for post-COVID-19 syndrome.

**Condensed abstract:**

This review examined cardiac function, vascular function, blood biomarkers and physical activity in patients post-COVID-19. Evidence suggests long-term dysfunction in multiple organ systems and exercise intolerance due to various factors, including endothelial damage and, in some patients, subclinical ventricular dysfunction. We highlight knowledge gaps for further research to aid post-COVID-19 rehabilitation.

## Key perspective

- Patients post-COVID-19 may exhibit sequelae in multiple organs, contributing to low cardiorespiratory fitness and exercise intolerance. Imaging evidence shows persistent impaired micro- and macrovascular endothelial function and mostly subclinical cardiac involvement in patients post-COVID-19, which might be related to these challenges.
- The exact mechanisms and long-term effects of these cardiovascular changes remain unclear, indicating a need for further research to fully understand and address these post-COVID-19 health challenges.
- The findings have implications for clinical practice, as they inform the development of targeted rehabilitation programs for patients post-COVID-19.

Rehabilitation for patients with post-COVID-19 syndrome demands an individualized approach, where structured exercise, though beneficial for some, may not be suitable or desirable for all. Taking into account the individual’s health conditions and recovery trajectories is vital.

## Introduction

Unmatched epidemiologic and clinical research efforts have provided rapid information on the risk factors, presentation, pathophysiology and treatment of the Coronavirus disease 2019 (COVID-19).^1^ However, even if hospitalized patients receive state-of-the-art treatment, including acute rehabilitation, many remain weakened due to residual symptoms.^2^ A review of 52 studies found that 29.3% of patients reported fatigue and 19.6% reported dyspnoea 1–4 months post-hospitalization, with 75% experiencing at least one persistent symptom at 8–12 months.^3^ Additionally, a larger review of 194 studies with 735,006 participants revealed that, on average, 45% of COVID-19 survivors experienced at least one persistent symptom approximately 4 months postinfection, with fatigue being common across both hospitalized and non-hospitalized cohorts.^4^ In general, population-based studies show a lower prevalence. For instance, Swiss data indicate that 22.9%, 18.5% and 17.2% of all adults post-COVID-19 reported not returning to their normal health status before infection at 6, 12 and 24 months post-infection, respectively.^5^ Furthermore, a meta-analysis of 53 studies found that 6.2% of individuals globally experienced at least one of three self-reported long-COVID symptom clusters at 3 months postinfection.^6^ Nonetheless, these studies dramatically show the burden affecting patients with persistent sequelae of COVID-19 (post-COVID-19 syndrome).

Exercise intolerance, an umbrella term for impaired cardiorespiratory fitness (CRF) and physiological as well as psychological symptoms of exhaustion (i.e. dyspnea, fatigue, dizziness), is a key complaint in post-COVID-19 syndrome.^7^ Deconditioning is often understood as a decreased CRF due to inactivity and has been suggested as a potential cause.^8,9^ However, this explanation is overly simplistic.^7^ The causes of this exhaustion extend beyond mere deconditioning.^7^ Our previous review of 26 studies discussed the contribution of different organ systems and deconditioning to low CRF based on cardiopulmonary exercise testing data.^7^ We highlighted that both peripheral and cardiovascular factors, not attributable to deconditioning alone, may contribute to exercise intolerance in these patients.^7^ Although cardiopulmonary exercise testing is a crucial tool for assessing the integrative responses of the pulmonary, cardiovascular and peripheral organ systems during exercise, it may not capture the microlevel pathophysiological processes contributing to exercise intolerance. An in-depth synthesis of this evidence that goes beyond the findings of cardiopulmonary exercise testing is crucial to uncover the particular mechanisms involved in exercise intolerance. Behavioral aspects are also worth considering because of their close coupling with CRF and also exercise intolerance.

We thus aim to provide a narrative review of key publications examining the medium- to long-term status (⩾3 months post-infection) of the peripheral and cardiovascular organ systems, along with pertinent blood biomarkers, which may contribute to exercise intolerance and low CRF post-COVID-19.

## Review of relevant literature

A narrative review of the literature was performed. We searched the databases Medline, EMBASE (on Ovid) and COVID-19 LOVE by Epistemonikos for publications, including adults post-COVID-19 that reported data on the following parameters:

- CRF
- Cardiac function
- Macro- and micro-vascular function
- Blood biomarkers of disease-specific impairment and endothelial dysfunction
- Lipidome
- Physical activity

Adults post-COVID-19 were defined as individuals ⩾18 years of age considering themselves recovered or those with post-COVID-19 syndrome. This review focused on studies with medium- to long-term follow-ups of >3 months post-acute COVID-19. Studies in languages other than German or English were excluded. In addition to original articles, pre-prints, reviews and meta-analyses were considered eligible and have been highlighted accordingly. Given the variability in study populations, we differentiate between ‘patients post-COVID-19’ and ‘patients with post-COVID-19 syndrome’ to provide clarity:

- Patients post-COVID-19: This term refers to all individuals who have had a confirmed COVID-19 infection, irrespective of their current symptom status. This group includes both those who have fully recovered and those who may still experience residual symptoms. We use this term when the studies do not specifically distinguish between individuals with and without ongoing symptoms.
- Patients with post-COVID-19 syndrome: This term is used exclusively for individuals who continue to experience persistent symptoms beyond the acute phase of the infection. These patients meet the criteria for post-COVID-19 syndrome, which is characterized by ongoing symptoms that were present in 100% of the patients in these studies.

If differentiation between these groups was not possible, we additionally provided information on symptom prevalence at follow-up in tables or text, wherever available. Of note is that the definition of post-COVID-19 syndrome is not uniform across original studies, particularly regarding the duration of symptoms. Symptoms can be continuous, relapsing or remitting.

## Results

### Cardiac function

SARS-CoV-2 can potentially damage the heart both directly (e.g. endothelial injury or angiocentric macrophage-driven inflammatory processes, distinct from classical anti-viral inflammatory responses^10^) and indirectly (e.g. hypoxia, a hypercoagulable state and its induction of a detrimental cytokine storm^11^) leading to structural und functional cardiac malfunction. Numerous studies have investigated cardiac involvement during acute COVID-19 in hospitalized patients with a prevalence of 5%–38%, increasing with disease severity.^12,13^ Acute cardiac manifestations include myocardial and/or pericardial inflammation, left ventricular (LV) dysfunction, reduced left ventricular global longitudinal strain (LVGLS), diastolic dysfunction, heart failure with preserved or reduced ejection fraction, cardiogenic shock, myocardial infarction, Takotsubo syndrome, arrhythmias and sudden cardiac death.^11,14^ Most studies investigating cardiac involvement focus on acute COVID-19, while data about the post-acute and chronic state after apparent COVID-19 recovery is lacking.^11,15^ Moderate- to long-term cardiac sequelae of COVID-19 remain insufficiently explored. Our review of ten studies involving 828 patients who had mild-to-severe COVID-19 and underwent follow-up cardiac imaging (echocardiography or cardiac MRI (cMRI)) summarizes what is known so far about the medium- to long-term cardiac sequelae of COVID-19. Twelve initially screened studies were excluded due to their short-term follow-up duration (<3 months). An overview of the included publications is provided in Table 1, where studies are sorted from top to bottom according to the period between acute COVID-19 and follow-up (from medium-term (i.e. 3-months post) to long-term (i.e. 1-year post)).

**Table 1.** Echocardiographic findings in ascending order according to the time of assessment after infection.

Author	Country	Population *n* (% female), mean (SD) age	Acute disease severity	Time of assessment after infection	Echocardiographic findings	cMRI findings
Moody et al.^16^	England	P: 79 (26%), 57 (11) years	Hospitalized with pneumonia, persistent symptoms: unknown	3 months after hospitalization	- 71% with normal TTE (vs 46% during hospitalization) - Persistent adverse ventricular remodeling in 29% - RV adverse remodeling (20%), LV adverse remodeling (6%), biventricular involvement (3%)	N/A
Akkaya et al.^17^	Turkey	P: 105 (40%), 45 (13) years. C: 105, age- and sex-matched, healthy	Non-hospitalized, mild disease persistent symptoms: unknown	3 months after diagnosis	- Subclinical RV dysfunction: decrease in RVGLS and RVFWLS parameters - RV diameter, systolic pulmonary artery pressure and RV myocardial performance index higher than in controls - TAPSE, RV fractional area change and RV S’ lower than in controls	N/A
The Writing Committee for the COMEBAC Study Group et al.^20^	France	P: 83 (N/A%), N/A years	ICU or symptomatic patients only persistent symptoms: 51% with >1 symptom since COVID-19 (fatigue 31.1%, memory difficulties 17.5%, dyspnea 16.3%, persistent paresthesia 12.1%)	4 months	Low prevalence of LV dysfunction (LVEF < 50% in only 9.6%, only ICU patients)	N/A
Lambadiari et al.^19^	Greece	P: *70 (37%), 55 (9) years. C1: 70, age- and sex-matched, with untreated hypertension. C2: 70, age-, sex-and risk factor-matched, healthy	- 34% mild (no hospitalization) - 33% moderate 33% severe. Persistent symptoms: 38% with post-infection symptoms (fatigue 15.7%, dyspnea 12.8%, cough 4.3%, chest pain 4.3%)	4 months	- Impaired LVGLS, RVGLS, RVFWS - TAPSE and RV S’ lower than in controls - No association with disease severity - (Published in paper by Ikonomidis, Lambadiari^25^*: higher myocardial global wasted work (GWW), decreased myocardial global work efficiency (GWE) compared to controls)	N/A
Dennis et al.^22^	England	P: 201 (71%), 44 (11) years. Post-COVID-19 symptoms. C: 36, age-matched, healthy	19% hospitalized; Persistent symptoms: 100% with post-COVID-19 syndrome (fatigue 98.0%, dyspnea 88.0%, muscle ache 86.5, headache 82.5%, joint pain 78.0%, chest pain 73%, cough 73.0%, fever 72.0%, sore throat 71.5%, diarrhea 59.0%, abnormal pain 54.0%, wheezing 49.0%, inability to walk 40%, runny nose 34.0%)	141 days after symptom onset	N/A	- 19% myocarditis - 9% systolic dysfunction
Pelà et al.^18^	Italy	P: 160 (40%), 60 (12) years	73% hospitalized (15% ICU). Persistent symptoms: dyspnea 70.0%, cough 9.0%, fatigue 57.0%, chest pain 28.0%, palpitations 29.0%, myalgia 13.0%, sleep-wake cycle alterations 50.0%	5 months after discharge from hospital or outpatient clinic	RV dilation, increased pulmonary resistance, bi-ventricular systolic-diastolic dysfunction; for example, impaired relaxation (reduced e’/a’ and e’ wave)	N/A
Raafs et al.^21^	Netherlands	P: 42 (31%), 64 (13) years	ICU patients. Persistent symptoms: 71.0% > 1 cardiac symptom (chest pain 21.0%, palpitations 14.0%)	6 months after hospitalization	GLS abnormal in 24% (subclinical LV dysfunction, normal LVEF)	(Sub-sample: *n* = 38) - 42% with cMRI abnormalities - GLS abnormal in 12% - 21% with late gadolinium enhancement (3 patients with myocarditis, 3 patients with pericarditis)
Ikonomidis et al.^25^	Greece	P: *70 (37%), 55 (9) years. C: 70, age-, sex-and risk factor-matched, healthy	34% mild (no hospitalization) 33% moderate 33% severe 38% with post-infection symptoms at 4 months follow-up	1 year	- Significant improvement of RVGLS, RVFWS and TAPSE since 4-months follow-up; no difference to controls - “Borderline improvement” of LVGLS since 4-months follow-up, but still impaired compared to controls - Modest improvement of GWW and GWE since 4-months follow-up, but remaining impairment versus control group	
Luchian et al.^23^	Belgium	P: 66 (approx. 40%), 51 (11) years	Hospitalized patients, 23 patients with persistent dyspnea (35%) without pre-existing cardio-respiratory diseases	1 year after hospitalization	Persistent dyspnea was associated with lower LVGLS, global constructive work and global work index → subclinical cardiac dysfunction	N/A
Wood et al.^24^	Denmark	P: 22 (86%), Mdn: 47 (IQR 41, 53) years. Post-COVID-19 symptoms. C: 22, age-and sex-matched, healthy	Persistent cardiac symptoms (100%); acute disease: 4 patients in hospital for >24 h, 1 ICU	382 days (median) since diagnosis	No evidence for significant cardiac abnormalities	No evidence for significant cardiac abnormalities

C: controls; cMRI: cardiac magnetic resonance imaging; GWW: global wasted work; GWE: global work efficiency; ICU: intensive care unit; Mdn: median; LV: left ventricle; LVEF: left ventricular ejection fraction; P: patients; RV: right ventricle; RV S’: right ventricle systolic function; RVFWLS: right ventricle free-wall longitudinal strain; RVGLS: right ventricle global longitudinal strain; TAPSE: tricuspid annular plane systolic excursion; TTE: transthoracic echocardiogram.

* Same cohort, follow-ups at 4- and 12-month post COVID-19.

Medium-term echocardiographic follow-up (3 months) revealed persistent adverse ventricular remodeling, especially of the right ventricle (RV) (20%), in 79 previously hospitalized patients with COVID-19-induced pneumonia.^16^ Also, another study found subclinical RV dysfunction, increased RV diameter and pulmonary resistance even in non-hospitalized patients 3 months after mild COVID-19.^17^ Of note, the symptom burden at follow-up was not reported. An Italian cohort of 160 mostly hospitalized (73%) patients revealed RV dilation, impaired relaxation and increased pulmonary artery pressure 5 months after hospital discharge (51% ⩾1 symptom at follow-up).^18^ Lambadiari et al.^19^ found both RV and LV subclinical impairment in patients at 4-month follow-up, regardless of disease severity (38% with symptoms at follow-up). LV dysfunction (9.6% of 83 patients^20^) and remodeling (6% of 79 patients^16^) were generally observed in a smaller proportion of patients, even if they had been treated in the intensive care unit (ICU) for severe disease. However, subclinical LV dysfunction (abnormal LVGLS) was present in 24% of patients of a smaller Dutch cohort (*n* = 42, 71% with symptoms at follow-up) 6 months post-ICU hospitalisation.^21^ The authors also found 42% of their patients to present with cMRI abnormalities, including signs of myo- and pericarditis.^21^ Dennis et al.^22^ investigated patients with post-COVID-19 syndrome 4.5 months after acute infection reporting signs of myocarditis and systolic dysfunction in 19% and 9%, respectively. Only three studies reported follow-up findings ⩾1 year after acute COVID-19.^23–25^ Luchian et al.^23^ found over one-third (35%) of their cohort to suffer from persistent dyspnea even though they had no pre-existing cardio-respiratory diseases. On echocardiographic examination, this was associated with lower (but normal range) LVGLS, global constructive work and global work index compared to patients without persistent dyspnoea.^23^ Another study found RV function to be significantly improved after 12 months compared to their 4-month follow-up (38% with symptoms at follow-up),^19^ while LVGLS and myocardial work remained impaired (unknown symptom prevalence at 12 months).^25^ In a smaller cohort (*n* = 22) of patients with persistent cardiac symptoms (100%), there was little radiological evidence for cardiac abnormalities.^24^

### Micro- and macrovascular function

COVID-19 is considered both a micro-vascular and macro-vascular endothelial disease.^26^ This is evident by numerous studies performed in patients with acute COVID-19 reporting impaired endothelial function.^27,28^ Yet, less is known about the period post-acute-COVID-19. In this section, we will thus review the key studies with moderate- to long-term follow-ups and distinguish between micro- and macro-vascular endothelial function.

Several non-invasive biomarkers exist to diagnose microvascular dysfunction. The high potential of retinal vessel imaging to quantify systemic microvascular dysfunction non-invasively has recently been highlighted by the European Society of Cardiology.^29^ Especially retinal arteriolar narrowing and venular widening have been associated with higher cardiovascular mortality risk^30^ and long-term cardiovascular outcomes.^31,32^ Interestingly, retinal arteriolar and venular vessel diameters seem to be elevated in acute COVID-19 patients.^33–36^ Studies that included patients with different disease severities showed that vessel widening appears to depend on severity.^34,35^ Only a few studies (*n* = 4; 157 patients) investigated retinal vessel diameters in patients post COVID-19 (see Table 2). Invernizzi et al.^35^ reported wider arteriolar and venular retinal vessel diameters in 32 patients with severe COVID-19 6 months after hospitalisation compared to 53 unexposed healthy individuals. Interestingly, the acute wider arteriolar and venular vessel diameters of patients with non-severe COVID-19 were normalized 6 months later.^35^ Less is known about the long-term consequences of COVID-19-induced widening of arterioles and venules.

**Table 2.** Retinal imaging findings in ascending order according to the time of assessment after infection.

Author	Country	Population *n* (% female), mean (SD) age	Acute disease severity	Time of assessment	Assessment method	Main findings
Aşıkgarip et al.^33^	Turkey	P: 25 (64%), 34 (20) years. C: 25 healthy (44%), 35 (19) years	Hospitalized due to COVID-19	At baseline and 4 months after remission	Optical coherence tomography	- Significantly increased diameters of the temporal and nasal retinal arteries and veins in patients compared to healthy controls at baseline - At 4-month, significant decrease of retinal vessel diameters in all quadrants compared to baseline - No significant difference between the follow-up measurements of patients after and healthy controls
Invernizzi et al.^34^	Italy	P: 54 (30%), 50 (16) years, C: 133 (50%) unexposed subjects, 44 (13) years	Acute COVID-19. Exclusion: previous intensive care unit stay and known retinal disorders (i.e. diabetic retinopathy)	Within 30 days of onset of systemic symptoms	Digital Retinography System (DRS) fundus camera	- Significantly larger mean arterial and venular diameters in patients than controls - Venular diameter was larger in more severe COVID-19 and inversely associated with time to symptoms onset
Invernizzi et al.^35^	Italy	P: 59 eyes from 32 (31%) patients, 48 (14) years. C: 80 eyes from 53 (57%) unexposed, 44 (13) years	Patients admitted to the Infectious Diseases Department of a tertiary referral center diagnosed with acute COVID-19 (positive nasopharyngeal swab and symptoms onset within 30 days before the fundus screening)	Second part of study describing follow-up findings at 6 months (30 days) from the first fundus examination	Digital Retinography System (DRS) fundus camera	- Mean arterial and venular diameters significantly decreased in patients at follow-up - Yet, vessels diameter remained significantly higher in patients with severe COVID-19 than unexposed controls
Aydemir et al.^36^	Turkey	P: 46 (54%), 38 (10) years. C: 38 (50%) age- and sex-matched healthy, 40 (8) years	Patients successfully treated for COVID-19. Only patients who received anti-viral and anti-coagulation treatments were included. None of the patients were admitted to the intensive care unit	1 month after complete recovery	Fundus camera system: best-corrected visual acuity (BCVA), slit-lamp bio-microscopy, intraocular pressure (IOP) values with Goldman applanation tonometry and posterior segment examination with dilated pupillary	Larger retinal arteries and venules in patients than healthy controls

C: controls; P: patients.

On the macro-vascular level, flow-mediated dilation (FMD) is the non-invasive gold standard for measuring endothelial function.^37^ Ambrosino et al.^28^ meta-analyzed 12 studies with 644 patients up to 1 year post-COVID-19 and 662 controls. They found impaired endothelial function measured with FMD in patients compared to controls.^28^ This was also observed when studies involving patients with cardiovascular disease were excluded.^28^ The difference persisted when looking at studies with follow-ups of more than 3 months isolated, and more considerable impairments were seen with a higher prevalence of patients with post-COVID-19 syndrome.^28^ Likewise, another meta-analysis reported impaired endothelial function in patients post-COVID-19.^27^ In both meta-analyses, endothelial function remained significantly impaired with increasing follow-up time, although gradual improvement may occur.^28,27^ This is further supported by more recent longitudinal data of Ikonomidis, Lambadiari et al.^25^ No significant difference in FMD was apparent in patients post-COVID-19 between 4-month and 12-month follow-ups.^25^

### Blood biomakers of disease-specific impairment and endothelial dysfunction

A meta-analysis highlighted key blood biomarkers in COVID-19 survivors with and without post-COVID-19 syndrome.^38^ A high percentage of survivors with post-COVID-19 syndrome had a still raised C-reactive protein (817/1581, 51.7%), D-dimer (784/1683, 46.6%), lactate dehydrogenase (736/1549, 47.5%) and leukocytes (436/949, 45.9%).^38^ These biomarker elevations (D-dimer, lactate dehydrogenase, lymphocytes) correlated with organ abnormalities, lactate dehydrogenase and leucocyte elevation was linked to <6 months post-COVID-19, while D-dimer was rather associated with ⩾6 months post-COVID-19.^38^

Furthermore, Hartung et al.,^39^ found that in 969 patients post-COVID-19 matched to COVID-19-free controls, 77% of patients >6 months post-infection still had increased C-reactive protein along with 19% being fatigued, compared to only 8% of controls with no C-reactive protein elevation. 26% of these patients had mild and 1% moderate cognitive impairment.^39^

Elevated C-reactive protein during acute COVID-19 also predicted neurological complications 9–13 weeks post-COVID-19.^40^ Moreover, 42 patients were studied 3–6 months post-COVID-19 and 4% of them had a persistent inflammatory response evident by elevated C-reactive protein, while 17% showed increased angiotensin-converting enzyme 2 concentrations.^41^

Peluso et al.^42^ investigated 280 patients post-COVID-19 where 74.2% showed persistent symptoms at a median of 4 months post-COVID-19 and 34.6% of them had even >5 symptoms such as fatigue and neurocognitive dysfunction. Symptoms were independently associated with serological evidence suggesting recent Epstein–Barr virus, but not cytomegalovirus reactivation.^42^

In another study, 50% of convalescent patients had ⩾1 cardiovascular risk factor.^43^ They showed more endothelial injury measured by circulating endothelial cells than healthy controls independent of cardiovascular risk.^43^ Proinflammatory interleukin (IL)-1β, IL-17A, IL-2 and RANTES (Regulated and Normal T cell Expressed and Secreted) were significantly higher in patients post-COVID-19 with cardiovascular risk than those without during the early convalescent phase (median 7 days post hospital discharge).^43^ Regeneration by circulating endothelial progenitor cells was not different between the groups,^43^ but 3 months after overcoming COVID-19, the presence of vascular sequelae in patients post-COVID-19 could be detected by an abnormal increase in the number of circulating endothelial progenitor cells in cell culture (endothelial colony forming cells) compared to controls (2.81 (2.33) vs 1.23 (1.86), *p* = 0.001).^44^

### Lipidome

Bioactive lipids are involved in many essential cellular processes, including cell growth, cell adhesion and migration, senescence, apoptosis, inflammation, immune response and angiogenesis.^45,46^ Therefore, it is not surprising that lipids play an essential role in cardiometabolic health and diseases.^45^ Recently, risk assessment models, including levels of specific lipid species and their ratios, were proven to predict cardiometabolic outcomes more accurately and precisely than traditional cardiovascular risk factors (i.e. high blood pressure, high cholesterol, smoking) in patients with and without coronary artery disease.^47,48^ As the pathological mechanisms underlying COVID-19 involve endothelial and cardiometabolic dysfunctions, investigating lipid metabolism is not only of pathophysiological interest but could also improve patients’ stratification and prediction of severe evolution.^49,50^

Two preliminary small sample studies (46 and 59 individuals with COVID-19) revealed significant changes in fatty acid, serum sphingolipid, glycerophospholipid and glycerolipid species in acute COVID-19.^51,52^ Further, these studies indicated a potentially active role of sphingolipids in SARS-CoV-2 infection and disease severity as the virus-specific molecular targets angiotensin-converting enzyme and transmembrane serine protease 2, which are embedded in the sphingolipid-enriched lipid raft.^51^ A subsequent small sample-sized study assessed the lipid profile in 19 convalescent patients post-COVID-19 and found unsaturated fatty acids to be continuously altered compared to a pre-pandemic cohort.^53^

### Physical activity

Available studies on patients post-COVID-19 are primarily based on self-reported physical activity (PA) data assessed by questionnaires (Table 3). Patients either filled out well-established (International Physical Activity Questionnaire, *n* = 1)^54^ or newly developed tools (*n* = 2)^55,56^ allowing for an estimation of PA. Three studies assessing patients with post-COVID-19 syndrome found that PA levels are generally low,^54–56^ negatively correlate with disease severity (mild vs moderate),^54^ but tend to increase over time (3–6-month follow-up) while remaining below reported pre-COVID-19 levels at 6-month follow-up.^55^ One study used the Rating of Perceived Capacity Scale to indicate the patients’ perceived aerobic capacity.^56^ All included patients suffered from post-COVID-19 and reported low perceived CRF (median rating = 5 out of 20, indicating that walking or cycling slowly was the most strenuous activity that could be sustained for >30 min). Accelerometer-based assessment of PA was only used in one sizeable study of hospitalized patients.^58^ The authors reported hospital survivors of COVID-19 having low levels of PA.^58^ More severe illness was associated with lower levels of moderate-to-vigorous and total PA, confirming what has been observed in some survey-based studies.^54–56,58^

**Table 3.** Findings of physical activity assessment in ascending order according to the time of assessment after infection.

Author	Country	Population n (% female), mean (SD) age	Acute disease severity	Time of assessment	PA assessment method	Main findings
Ekstrand et al.^57^	Sweden	P: 766 (89%), 48 (10) years. Post-COVID-19 symptoms	Majority non-hospitalized (89%)	>2 months after infection (mean 13 months)	- Survey (self-reported): perceived aerobic capacity (Rating of Perceived Capacity Scale: 1–20)	- Median perceived aerobic capacity measured was 5 (IQR 3–7), that is. walking or cycling slowly was the most strenuous activity that could be sustained for >30 min
Tanriverdi et al.^54^	Turkey	P: 48 (54%), 39 (8) years. Post-COVID-19	Mild or moderate disease (39% hospitalized, no ICU)	>2 months since symptom onset (mean 144 days)	- Survey (self-reported): International Physical Activity Questionnaire (IPAQ) - Estimation of physical activity level (PAL) as low (<600 MET min/week), moderate (600–1500 MET min/week) and high (>1500 MET min/week)	- Significant proportion of post-COVID-19 patients had low PALs, especially those with moderate disease (vs mild) - Low PAL in 28% of the mild and 52.2% of the moderate disease severity group - Moderate PAL in 32% of the mild and 34.8% of the moderate disease severity group - High PAL in 40% of the mild and 13% of the moderate disease severity group
Delbressine et al.^55^	Netherlands, Belgium	P: 239 (83%), Mdn: 50 (range 39–56) years. Post-COVID-19 symptoms	26% hospitalization, no ICU	3 and 6 months after symptom onset	- Survey (self-reported): performance of different types of physical activity/sports, besides (yes/no)	- 3 months: fewer activities compared to pre-COVID-19; almost 44% not able to be physically active or perform sports/activities due to COVID-19 - Significantly decreased weekly walking time after 3 months of follow-up (60 (15–120) min vs pre-COVID-19: 120 (60–240) min/week; *p* < 0.05) - 6 months follow up: walking time was still significantly lower compared to pre-COVID-19 but significantly increased compared to 3 months follow-up (3 months: 60 (15–120) min vs 6 months: 90 (30–150) min; *p* < 0.05)
Plekhanova et al.^58^	England	P: 715 (35%), 58 (14) years (♀), 60 (12) y (♂). C1: 685, type 2 diabetes, similar sex and age. C2: 232, office workers, similar age	Hospitalized, different severity clusters	245 days (median) after hospitalization	- Accelerometry (GENEActiv)	- Low levels of PA in hospital survivors of COVID-19 (mean (SD) of 14.9 (4.7) min/day (♀) and 21.0 (22.3) (♂) of MVPA) - More severe acute illness was associated with lower total activity and MVPA
Tabacof et al.^56^	USA	P: 156 (69%), Mdn: 44 (range 13–79) years. Post-COVID-19 symptoms	Majority non-hospitalized (89%)	351 days (82–457) after infection	- Survey (self-reported): author-developed tool to screen for regularity of MVPA (150 min/week*)	- Patients were completing regular physical activity* less frequently after COVID-19 infection (asked separately about moderate and vigorous intensities) - Physical activity was the most common trigger for symptom exacerbation (86%)

C: controls; ICU: intensive care unit; IQR: interquartile range; Mdn: median; MET: metabolic equivalents of task; MVPA: moderate-to-vigorous physical activity; P: patients; PA: physical activity; PAL: physical activity level.

## Discussion

The medium- to long-term health status of the peripheral and cardiovascular organ systems, along with pertinent blood biomarkers and their potential contribution to exercise intolerance and low CRF post-COVID-19 are discussed in the respective sections below.

### Cardiac function

We found that available studies on cardiac sequelae are mainly limited by their short follow-up duration. The available data suggest that cardiac involvement, especially of the LV, might be persistent in some patients with and without post-COVID-19 and that although functional impairments may be subclinical, they might partly explain persisting symptoms such as exercise intolerance.^59^ However, hearts that appear normal or exhibit subclinical abnormalities at rest may function differently during exercise, where limitations often occur. In support of this, D’Andrea et al.^60^ (*N* = 370) found frequent biventricular involvement with impaired LV and RV systolic function, yet often missed by conventional indices like EF and TAPSE. These could however be detected by more sensitive Speckle-Tracking Echocardiography indices.^60^ The authors concluded that the reported alterations were functionally relevant and associated with reduced exercise time, higher prevalence of pulmonary hypertension and greater pulmonary congestion during exercise.^60^ This discrepancy highlights the importance of evaluating cardiac function also during exercise to fully understand the contribution of cardiac changes to exercise intolerance. More details on stress echocardiography in patients post-COVID-19 are provided elsewhere.^61^

### Micro- and macrovascular function

As described above, retinal vessel diameters seem to be affected by COVID-19 in acute and midterm settings. However, less is known about the long-term effects of COVID-19 on retinal vessel diameters as systemic microvascular health marker. Several potential mechanisms may be responsible for arteriolar and venular widening in patients with acute COVID-19 infection. Previous publications have reported that a high inflammation status is associated with wider retinal venules.^62^ In addition, a low oxygen partial pressure might be a further mechanism for why retinal vessel diameters are wider in patients with COVID-19 compared to healthy controls. Bosch et al.^63^ have shown that arteriolar and venular retinal vessel diameters are wider at high altitudes with a lower oxygen partial pressure. Other acute mechanisms might also directly or indirectly affect retinal vessel diameters, such as cardiac output, blood pressure, oxidative stress or endothelial dysfunction.^64^ More research is necessary to better understand the underlying mechanisms of retinal vessel widening in patients with acute COVID-19. Microcirculation in general is an important regulator of blood flow and therefore a gatekeeper of sufficient oxygen supply to various organs. In the context of exercise intolerance, microvascular dysfunction is an important limitation factor for exercise capacity.^65^ Mahfouz et al.^66^ showed that microvascular dysfunction was an independent predictor for reduced exercise tolerance in patients with heart failure. Our working group showed that CRF adjusted for age and sex explained 27% of retinal vessel diameter variance.^67^ However, less is known about the influence of microvascular dysfunction on CRF and exercise intolerance in patients with post-COVID-19 syndrome. Additionally, the underlying mechanisms of how COVID-19 affects microvascular function still need to be clarified. Therefore, collecting more data on retinal microvascular structure and function is important to get a deeper insight into COVID-19-induced retinal microvascular abnormalities, their underlying mechanisms as well as their influence on exercise intolerance in patients with post-COVID-19 syndrome.

Moreover, a large body of evidence highlights long-term macrovascular endothelial impairment, especially in patients with persistent symptoms. This may have important implications for exercise intolerance and low CRF. Several studies have suggested endothelial dysfunction as a pathogenic contributor to these.^68–70^ Ambrosino et al.,^68^ for instance, found FMD to be directly associated with CRF, peak end-tidal carbon dioxide pressure and inversely with ventilation/carbon dioxide production slope. The latter two markers are indicative of pulmonary vascular limitations.^68^ These findings are complemented by correlations observed between lung diffusion capacity and circulating endothelial cell markers, further highlighting the impact of endothelial health on pulmonary function.^71^ Moreover, other pathways through which endothelial dysfunction might contribute to low CRF and exercise intolerance should be investigated such as via impaired blood flow and oxygenation.^72^ This could aid the development of targeted therapies. There is also a pressing need for more extensive research focused on the long-term recovery of endothelial function, >12 months post-infection. Such studies would offer valuable information on the persistence and potential reversibility of COVID-19-related vascular changes. Lastly, assessing endothelial function in combination with other health parameters such as CRF, blood biomarkers or physical activity could be valuable to obtain a complete picture of patients’ health condition post-COVID-19.

### Blood biomakers of disease-specific impairment and endothelial dysfunction

Hematologic, biochemical, immune and vascular blood biomarkers have gained increasing attention as some have been shown to discriminate between those with non-severe and severe or even fatal COVID-19 courses^73^ or to predict the risk of post-COVID-19 syndrome.^74^ However, biomarkers discussed in the literature and their interactions still need to be better understood, studied in larger cohorts and observed over a more extended period after infection to better distinguish between effects that resolve over time or indeed manifest in chronic disease.^75^ In this regard, biomarkers of inflammation, hypercoagulability and myocarditis are essential, such as C-reactive protein, D-Dimer and leucocyte count. Some studies showed that >1 of these biomarkers stayed elevated after the acute COVID-19 in patients who developed post-COVID-19 syndrome.^38,39^ Others, however, did not find any difference from those without persistent symptoms after 12 months.^76^ Some of these biomarkers could be linked to exercise intolerance, for example, elevated C-reactive protein during acute COVID-19 or even ⩾6 months post-infection was related to increased neurological sequelae or fatigue.^40,39^ Also, low angiotensin-converting enzyme 2 levels were associated with reduced handgrip strength pointing to endothelial dysfunction as cause of muscle fatigue.^41^

Interestingly, recent Epstein–Barr virus reactivation increased the risk of post-COVID-19 syndrome, whereas an underlying cytomegalovirus infection was related to decreased odds.^77^ These differential effects of chronic viral co-infections on the probability of developing and mainly manifesting post-COVID-19 need further investigation.^77^ Cytokine-storm-induced organ damage,^78^ visible in liver, pancreatic or renal serology, could prevail even up to 24 months after acute infection in previously hospitalized individuals and may also drive exercise intolerance. Moreover, data on surrogate markers for damage and regeneration directly at the endothelial cell level are scarce but should be addressed. Circulating endothelial cells, which represent the degree of endothelial damage,^43^ were elevated in patients with acute COVID-19 compared to COVID-19-free subjects and were a good predictor for disease severity.^26,43^ Endothelial regeneration by circulating endothelial progenitor cells is disturbed by SARS-CoV-2-induced transcriptome-level aberrations.^79^ Recently, Poyatos et al.^44^ found an elevated endothelial progenitor cell production in post-COVID-19 syndrome, but if this indicated a prompt response of the patient to repair the damaged endothelium or reflect a post-infection injury that will persist in time is not yet known. Therefore, the ratio of the total and apoptotic circulating endothelial regenerative capacity-to-damage (endothelial progenitor cell to circulating endothelial cell ratio) may be a precise biomarker for quantifying endothelial dysfunction at the cellular level.^80^ This would verify any disease-specific impairment seen at the level of micro- and macro-vasculature and be helpful as a monitoring tool for rehabilitation programs, as defects could be identified the earliest possible.

### Lipidome

Altogether, there is a limited body of literature that specifically investigated changes at the molecular species levels in patients with acute or post-COVID-19 syndrome. There are some hints that lipidome alterations might be linked to acute COVID-19 as well as with long-term sequelae of SARS-CoV-2. Yet, the current evidence is insufficient to draw definitive conclusions on a possible contribution of the lipidome to post-COVID-19 symptoms and exercise intolerance. As lipids are essential biologically active macromolecules, further investigating lipidome changes occurring during the course of the disease could help to better understand the physiological limitations observed following COVID-19.

### Physical activity

The current knowledge about PA patterns post-COVID-19 is limited and mainly based on self-reported data. Compared to objective assessment, that is, using accelerometers, this data acquisition method has several limitations discussed elsewhere.^81^ Reduced PA and diminished CRF are associated with an increased risk for (re-)infection and severe COVID-19.^82,83^ Moreover, PA levels have been linked to cardiac^84^ and vascular function,^85^ both relevant for patients with post-COVID-19 syndrome as previously discussed. However, reduced PA may also reflect the level of impairment due to residual symptoms. This highlights the importance of PA assessment in these patients and the need for precise PA guidelines for patients suffering from impaired CRF after COVID-19 infection (considering the range, severity, frequency and duration of their symptoms).^86^ We emphasize that PA levels below normal should not immediately be seen as a call for PA promotion. They should, together with other health outcomes, rather be interpreted as an indication of an underlying limitation that might prevent the individual from being active. This calls for individualized problem-solving approaches. Advanced analytical approaches of device-based PA data may contribute to gasping the bigger picture in these patients (described elsewhere in detail^87,88^). Taken together, the critical role of PA in COVID-19 rehabilitation and the limitations of the currently available literature warrant a thorough, objective examination of PA levels in patients post-COVID-19.

PA and exercise yield a range of health benefits for longevity, prevention of chronic disease and disability, osteoporosis, as well as mental health.^89,90^ Several studies yield evidence for the effectiveness of exercise interventions to improve CRF and reduce symptom severity.^91–93^ Yet, as pointed out previously,^94^ the promotion of PA and exercise in patients post-COVID-19 must occur on an individual basis. Particular caution is required when symptom-flare-ups, for example, due to post-exertional malaise, are expected.^94^ There is a need for more research into this patient population. In the absence of such issues, intensity, volume and periodization of the regime should be adapted to the individual’s fitness level and symptoms.^94^

### Limitations

This review has several limitations. First, we did not use a systematic search strategy and may have missed relevant studies. Yet, we aimed to discuss critical studies of the six research areas, provide an overview of gaps in the literature, and how these can be addressed in an integral approach. Second, we focus on specific aspects that are important to consider in these patients. This review does not constitute an exhaustive summary of all systems possibly affected post-COVID-19.

## Conclusions

We highlighted and discussed relevant sequelae in peripheral and cardiovascular organ systems of patients with and without post-COVID-19 syndrome that could contribute to low CRF and exercise intolerance. Findings of persistent cardiac involvement are partly supported by often subclinical findings in echocardiography studies (see Table 1) and evidence from stress echocardiography. Impaired micro- and macrovascular endothelial function may be additional contributors to post-COVID-19 syndrome and exercise intolerance (see Table 2). While changes in the circulating lipidome may potentially influence endothelial and cardiometabolic function, caution is advised in drawing firm conclusions, due to limited evidence. Although several potential mechanisms have been identified, the physiological processes underlying the persisting health issues linked to post-COVID-19 syndrome are still not well understood.

Furthermore, our review reveals that only little is known about the health status of patients more than 1 year post-COVID-19. Thus, we do not know the percentage of patients post-COVID-19 who will benefit from rehabilitation to the extent that they regain their pre-infection level of physical and mental function or, in turn, the share of patients who will need to accept and aim for maintaining the new (impaired) health condition. The main limitations across current COVID-19 and post-COVID-19 syndrome literature in general, and most studies covering the aspects we addressed in this review, are their focus on the acute disease and short-to-moderate follow-up durations. Moreover, studies and analyses focusing on patients with post-COVID-19 syndrome, not pooling all patients with previous COVID-19, would contribute to shedding more light onto the underlying pathophysiology of this condition.

## Perspective

As a perspective addressing the findings and issues highlighted in this narrative review, we focus on two levels, (a) research and (b) rehabilitation.

(a) Research: The heterogeneity of the patient population poses a major difficulty. Numerous studies have shown that a universal treatment approach is obsolete in post-COVID-19 syndrome. Thus, diagnosis and treatment should be specific to the individual. Gentilotti et al.,^95^ for instance, demonstrated that various clinical phenotypes of post-COVID-19 syndrome could be identified. Such phenotypes may also be worth considering when investigating the underlying mechanisms of exercise intolerance and low CRF. Many studies examined patients post-COVID-19 with some reporting residual symptoms and some not (see Tables). This has undoubtedly enriched our understanding, but future research should focus on comparing different subgroups within this population paired with an extensive cohort characterization. This approach would offer a more nuanced and individualized perspective, enhancing our understanding of the syndrome’s varied manifestations. Furthermore, although PA and exercise provide numerous health benefits, their promotion in patients post-COVID-19 must be tailored. More studies are needed to evaluate the effectiveness of exercise interventions in this context, taking into account the risk of symptom flare-ups, such as post-exertional malaise.
(b) Rehabilitation: It is impossible to recommend an exercise rehabilitation regime that is feasible and effective for all patients. Our findings suggest possible underlying mechanisms of exercise intolerance and low CRF that could be tackled by structured exercise training.^65^ However, the unpredictable long-term health outcomes in patients with post-COVID-19 syndrome add another layer of complexity to rehabilitation planning. The variability in recovery levels necessitates a highly flexible and adaptive approach to rehabilitation (see Tables).^7,96^ This means preparing to offer exercise training for those capable of regaining their pre-infection functionality and tolerating such, as well as supportive care for individuals who may need to adapt to a new health baseline. Central to this approach is the adoption of a patient-centred model.^97^ In this model, rehabilitation goals should be collaboratively established with the patient, taking into consideration their unique health trajectory and potential for recovery. The promotion of PA and exercise in patients post-COVID-19 should be carried out on an individual basis, particularly with caution in cases where symptom flare-ups, such as post-exertional malaise, are expected.^94^
